# Infarction of the Corpus Callosum: A Retrospective Clinical Investigation

**DOI:** 10.1371/journal.pone.0120409

**Published:** 2015-03-18

**Authors:** Shen Li, Xin Sun, Yu-meng Bai, Hua-min Qin, Xiao-mei Wu, Xiao Zhang, Jukka Jolkkonen, Johannes Boltze, Su-ping Wang

**Affiliations:** 1 Neurology Department, Dalian Municipal Central Hospital affiliated to Dalian Medical University, 116033 Dalian, China; 2 Pathology Department, the Second Hospital of Dalian Medical University, 116027 Dalian, China; 3 Department of Clinical Epidemiology and Center of Evidence Based Medicine, the First Hospital of China Medical University, 110001 Shenyang, China; 4 Radiology Department, Dalian Municipal Central Hospital affiliated to Dalian Medical University, 116033 Dalian, China; 5 Neurology Department, Institute of Clinical Medicine, University of Eastern Finland, 70210 Kuopio, Finland; 6 Department of Cell Therapy, Fraunhofer Institute for Cell Therapy and Immunology, 04103 Leipzig, Germany; 7 Translational Centre for Regenerative Medicine, 04103 Leipzig, Germany; University Medical Center (UMC) Utrecht, NETHERLANDS

## Abstract

**Objectives:**

The aim of this study was to investigate patients with ischemic infarctions in the territory of the corpus callosum to advance our understanding of this rare stroke subtype by providing comprehensive descriptive and epidemiological data.

**Methods:**

From January 1, 2010 to June 30, 2014, all cases of acute ischemic stroke diagnosed by clinical manifestation and diffusion weighted imaging in Dalian Municipal Central Hospital were investigated. The patients presenting with corpus callosum infarctions were selected and further allocated into genu and/or body and splenium infarction groups. Proportion, lesion patterns, clinical features, risk factors and etiology of corpus callosum infarction were analyzed.

**Results:**

Out of 1,629 cases, 59 patients (3.6%) with corpus callosum infarctions were identified by diffusion weighted imaging, including 7 patients who had ischemic lesions restricted to the corpus callosum territory. Thirty six patients had lesions in the splenium (61.0%). Corpus callosum infarction patients suffered from a broad spectrum of symptoms including weakness and/or numbness of the limbs, clumsy speech, and vertigo, which could not be explained by lesions in corpus callosum. A classical callosal disconnection syndrome was found in 2 out of all patients with corpus callosum infarctions. Statistical differences in the risk factor and infarct pattern between the genu and/or body group and splenium group were revealed.

**Conclusion:**

Corpus callosum infarction and the callosal disconnection syndrome were generally rare. The most susceptible location of ischemic corpus callosum lesion was the splenium. Splenium infarctions were often associated with bilateral cerebral hemisphere involvement (46.2%). The genu and/or body infarctions were associated with atherosclerosis. The most common cause of corpus callosum infarction probably was embolism.

## Background

The corpus callosum (CC) is the largest white matter tract in the human brain, interconnecting homologous association areas of both hemispheres with approximately 180 million callosal fibers passing through it [1]. The CC receives abundant blood supply from both the anterior and posterior cerebral circulation [2]. The rostrum and genu are supplied by the subcallosal and the medial callosal artery, respectively. Both vessels are derived from the anterior communicating artery. The pericallosal artery, a continuation of the anterior cerebral artery (ACA), gives rise to four branches providing the majority of blood supply to the CC body. The posterior pericallosal artery, a branch of the posterior cerebral artery (PCA), is a short penetrating arteriole providing blood supply to the splenium. There are anastomoses between the callosal branches of ACA and PCA near the splenium tip. Thus, isolated CC-supplying ACA branches or PCA occlusion does not necessarily result in an interruption of blood supply and subsequent infarction [3]. Hence, the CC infarction syndrome is relatively rare with only a few, mainly small-scale systematic clinical investigations being reported.

Damage to the CC usually produces disturbance of higher brain function. Giroud and Dumas described two classic symptoms of the CC infarction: (1) callosal disconnection syndrome including apraxia, agraphia, tactile anomia of the left hand, and alien hand syndrome (AHS) [4–6] as well as (2) frontal type gait disorders including a wide base, shuffling gait with short steps and loss of concomitant arm swing as the result of lacunar lesions in the anterior CC portion [5]. In this study, we used DWI in the early diagnosis of acute cerebral ischemia [7] to investigate the proportion, lesion patterns, clinical features, risk factors and etiology of CC infarction in order to advance the understanding of this specific and rare stroke subtype.

## Materials and Methods

### Ethics statement

Written informed consent was obtained from every patient on admission about any diagnostic and therapeutic procedure undertaken. Patients were also informed that non-personal information may be used for clinical investigations. The Dalian Municipal Central Hospital Ethics Committee approved the study. Since we applied a retrospective approach, all relevant information was taken from the clinical database of Dalian Municipal Central Hospital, but without re-informing the patients. However, since only anonymous data was used and patient’s privacy was not violated by this study, waiving post-hoc written informed consent to use data for scientific purposes was approved by the Ethics Committee (vote number 2014–012–01 from July 24, 2014). The study was conducted according to the principles expressed in the Declaration of Helsinki.

### Patients

We retrospectively reviewed 1,629 patients with ischemic stroke admitted to Dalian Municipal Central Hospital between January 1, 2010 and June 30, 2014.

### Imaging analysis and additional examinations

T2 and DWI images were obtained by a PHILIPS Achieva 3.0T magnetic resonance system with slice thickness of 6.1 mm after the patient was admitted to the hospital. Hyperintensity signals within the CC region in DWI (n = 59, 3.6% of study population) were considered to indicate CC infarction and Apparent Diffusion coefficient (ADC) values were routinely measured to determine the stage of stroke and for differential diagnosis.

Based on common anatomical understanding, three CC parts were discriminated: the genu, the body and the splenium. Another small part of the CC, the rostrum, was not included as a separate anatomic location since it could not be reliably distinguished by DWI. Patients were further assigned into two groups according to the location of the CC infarcts: patients with infarctions in the genu, body, or genu and body (referred hereafter genu and/or body group) (n = 23), being supplied by the anterior cerebral circulation and those with infarctions in the splenium (n = 26), which receives blood supply from the posterior cerebral circulation. We compared the proportion, lesion patterns, risk factors, and neurological dysfunctions between these two groups.

Magnetic resonance angiography (MRA, n = 14), transcranial doppler examination (TCD, n = 16), computed tomographic angiography (CTA, n = 14), digital subtraction angiography (DSA, n = 2) or carotid artery ultrasound (n = 51) were applied to assess intracranial and extracranial vessels. The method of choice was selected by the attending doctor based on patients’ general conditions including cardiac function, contrast agent allergies, and conscious state. Some patients were subjected to repeated vessel examination to receive a clear diagnosis and/or according to treating physician’s decision. Investigation for potential cardiac pathologies was conducted by electrocardiography (EKG, all patients), 24 hours continuous cardiac monitoring (n = 5), and transthoracic echocardiography (n = 45).

### Clinical assessment

Neurological examination comprised general examination, level of consciousness, mental state and higher cortical function examination, cranial nerve examination, motor function, sensory function, stretch reflex examination, meningeal irritation sign and autonomic nerve examination (all patients). The National Institute of Health (NIH) Stroke Scale on hospital admission and discharge were calculated. The median interval from symptom onset to admission was 42.9 (3–240) hours.

Next to information on gender, age and initial symptoms, individual risk factor profiles were carefully investigated. Assessed risk factors comprised hypertension, diabetes mellitus, dyslipidaemia (high density lipoprotein <0.80 mmol/L or low density lipoprotein >3.36 mmol/L), smoking, excessive drinking (>21 units per week for men, or >14 units per week for women [8]), prior stroke or transient ischemic attack (TIA), history of heart diseases (for instance, coronary heart disease, valvular disease, and atrial fibrillation) and other known cardiovascular pathologies. Atheromas of the intracranial and extracranial arteries were documented by MRA, TCD, CTA, DSA, and/or carotid artery ultrasound. We also screened for high sensitive C-reactive protein (hs-CRP) and homocysteine (HCY).

### Statistical analysis

DWIs were pseudonymously screened and reviewed in random order by investigators being unaware of patient’s identity. Clinical data were collected from electronic medical database and reviewed by trained neurologists.

Statistical analysis was performed using SPSS (version, 17.0). Student’s *t*-test was used for numerical variables of normal distribution, and the non-normally distributed numerical variables were analyzed with non-parametric test (Wilcoxon rank-sum test). Chi-squared test was used for categorical variables. A value of *p*<0.05 was considered statistically significant. All values were expressed as mean±standard deviation. A biostatistician was consulted to ensure appropriate statistical testing throughout the study.

## Results

**Fig 1:**
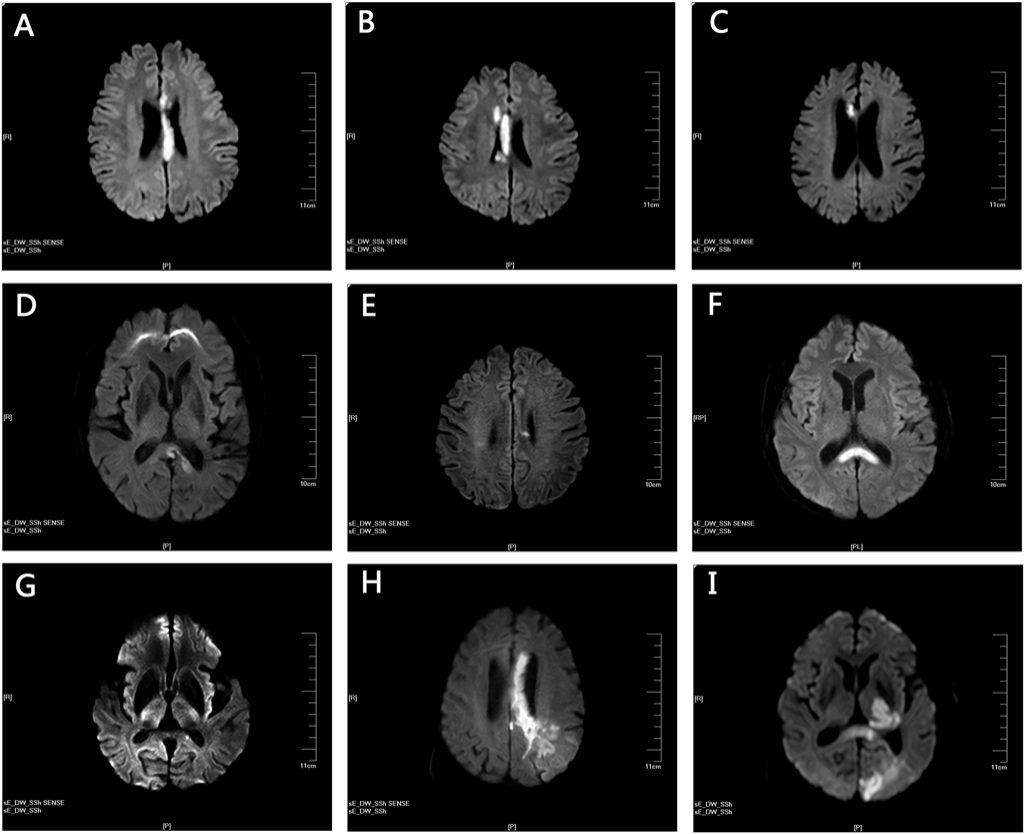
Representative images of pure and complex corpus callosum (CC) infarction. (A) to (G) shows all individual cases presenting with ischemic lesions restricted to the CC. (A) and (B) DWI showing infarctions in CC genu, body and splenium. (C) infarctions situated in the right body and genu of CC. (D) ischemic lesions were detected in the left splenium. (E) small infarction in the left CC body. (F) bilateral splenium infarctions. (G) very small infarction of the left splenium. (H) and (I) provide representative examples of patients showing additional ischemic lesions outside the CC. (H) infarctions involving the left CC genu and body as well as the occipital-parietal areas. (I) lesions located in the occipital lobe, the left thalamus as well as the left CC genu and body.

Fifty-nine CC infarction patients were included in the current study out of 1,629 stroke patient reviewed. The demographics, risk factors, neurologic dysfunctions, and location of CC infarcts are provided in Table 1. Among all stroke patients reviewed, unilateral strokes were seen in 40 patients. Twenty-six patients presented with infarctions in the left CC, 19 in the right CC and 14 with bilateral lesions. The ischemic damage was restricted to the CC territory (‘pure’ CC infarction) in 7 patients (Fig. 1A—G) while 52 patients presented with additional lesions apart from the CC (Fig. 1H, I). The most susceptible location was the splenium (n = 26+10), followed by genu (n = 8+8+10) and body (n = 7+8+10). Eight patients presented infarctions in both genu and body while genu, body and splenium were lesioned in 10 patients. No patient was diagnosed with body and splenium infarction.

**Table 1:** Demographics, risk factors, neurologic symptoms, and location of CC infarction.

	CC infarction	Genu and/or body infarction	splenium infarction	*p* value (genu and/or body infarction versus splenium infarction)	genu	body	genu and body	genu and body and splenium
Cases,n (%)	59 (100)	23 (40.0)	26 (44.1)		8 (13.5)	7 (11.9)	8 (13.6)	10 (16.9)
Age	68.4±11.5	67.9±11.7	70.6±10.6	0.398	71.3±10.4	64.6±15.5	67.4±9.5	63.7±12.8
Gender (male), n (%)	35 (59.3)	12 (52.2)	15 (57.7)	0.778	3 (37.5)	4 (57.1)	5 (62.5)	8 (80.0)
Risk factors, n (%)								
Hypertension	44 (74.5)	18 (78.3)	19 (73.1)	0.748	7 (87.5)	5 (71.4)	6 (75.0)	7 (70.0)
Diabetes mellitus	29 (49.2)	11 (47.8)	15 (57.7)	0.572	4 (50.0)	3 (42.9)	4 (50.0)	3 (30.0)
Dyslipidemia	32 (54.2)	12 (52.2)	15 (57.7)	0.778	5 (62.5)	3 (42.9)	4 (50.0)	5 (50.0)
Heart diseases	21 (35.6)	11 (47.8)	8 (30.8)	0.254	4 (50.0)	2 (28.6)	5 (62.5)	2 (20.0)
Excessive drinking	15 (25.4)	6 (26.1)	6 (23.1)	1.000	2 (25.0)	2 (28.6)	2 (25.0)	3 (30.0)
Current smoking	20 (33.9)	8 (34.8)	8 (30.8)	1.000	3 (37.5)	3 (42.9)	2 (25.0)	4 (40.0)
Prior stroke or TIA	14 (23.7)	5 (21.7)	8 (30.8)	0.532	0 (0)	3 (42.9)	2 (25.0)	1 (10.0)
Family history of stroke	2 (3.4)	1 (4.3)	0 (0)	0.469	0 (0)	0 (0)	1 (12.5)	1 (10.0)
Atheroma of ICA	54 (91.5)	22 (95.7)	23 (88.5)	**0.000** **	8 (100)	7 (100)	7 (87.5)	9 (90.0)
Atheroma of vertebrobasilar artery	25 (42.4)	9 (39.1)	12 (46.2)		5 (62.5)	1 (14.3)	3 (37.5)	4 (40.0)
Neurologic manifestation, n (%)								
Hemiparesis	43 (72.9)	17 (73.9)	17 (65.4)	0.552	5 (62.5)	5 (71.4)	7 (87.5)	9 (90.0)
Sensory abnormality	19 (32.2)	4 (17.4)	11 (42.3)	0.071	1 (12.5)	1 (14.3)	2 (25.0)	4 (40.0)
Ataxia	6 (10.1)	3 (13.0)	3 (11.5)	1.000	1 (12.5)	0 (0)	2 (25.0)	0 (0)
Aphasia	10 (16.9)	6 (26.1)	2 (7.7)	0.125	2 (25.0)	1 (14.3)	3 (37.5)	2 (20.0)
Dysarthria	13 (22.0)	4 (17.4)	7 (26.9)	0.506	1 (12.5)	1 (14.3)	2 (25.0)	2 (20.0)
Alien hand syndrome	2 (3.4)	0 (0)	0 (0)		0 (0)	0 (0)	0 (0)	2 (20.0)
Disturbance of consciousness	6 (10.1)	0 (0)	5 (19.2)	0.052	0 (0)	0 (0)	0 (0)	1 (10.0)
Admission time after the onset(hours)	42.9±47.0	44.0±36.8	44.2±59.1	0.240				
Bilateral cerebral hemisphere involvement	19 (32.2)	4 (17.4)	12 (46.2)	**0.039** *				
Initial NIH Stroke Scale score	3.0±3.7	2.7±3.1	3.1±4.3	0.691				
Discharge NIH Stroke Scale score	3.1±4.0	3.7±3.4	2.8±4.8	0.155				

Normally distributed numerical variables: Student’s *t*–test; non-normally distributed numerical variables: non-parametric test (Wilcoxon rank-sum test); categorical variables: chi-squared test.

*p<0.05.

TIA = Transient cerebral ischemia. ICA = internal carotid artery. Values are number (%) or mean±standard deviation

**Comparision of atheroma between genu and/or body and splenium group with their supplying artery respectively

According to the Trial of Org10172 in Acute Stroke Treatment (TOAST) classification [9], large-artery atherosclerosis was regarded to be the probable cause of stroke for the majority of the CC infarction patients (n = 34). Twenty-four patients were reported to have significant (n>50%) stenosis or occlusion of large arteries within the internal carotid artery (ICA) system, and 20 patients exhibited significant stenosis in the vertebrobasilar circulation. The most frequent risk factor was hypertension (n = 44), followed by dyslipidemia (n = 32) and diabetes mellitus (n = 29). Smoking was reported in 20 patients. A history of prior strokes or TIA was revealed in 14 individuals, excessive drinking was found in 15 patients. Abnormal hs-CRP values (>5mg/L) were observed in 8 out of 10 patients, while 11 from 28 patients were found to have abnormal HCY values (>15μmol/L). The diagnosis of stroke as a sequel of cardioembolism was made in 8 cases. There were 21 patients with known heart diseases and 12 individuals presenting cardiac embolic risk factors including hypokinetic left ventricular segment (n = 3), atrial fibrillation (n = 7), mitral value (n = 1), or mitral annulus prolapse (n = 2). A left atrial diameter ≥40mm was reported in 16 out of 45 patients subjected to transthoracic echocardiography. Five patients were diagnosed with stroke due to small-vessel occlusion. One patient was presented with acute stroke of other determined etiology and stroke in 11 patients was regarded to be of undetermined etiology.

In cases where CC infarctions coincided with additional, non-callosal lesions (herein termed ‘complex’ CC infarction, n = 52), clinical manifestations were diverse but mostly explainable by non-callosal lesions. Hemiparesis was the most frequent sign (n = 39), followed by sensory dysfunction (n = 17). Aphasia was seen in 11 patients and 11 patients experienced dysarthria. Five patients presented with confusion while visual field deficits were found in 2 patients. One patient was diagnosed with AHS and 3 individuals with top of basilar syndrome. Four patients experienced ataxia, 1 patient suffered from neglect and apraxia. The average NIH Stroke Scale score was 3.0±3.7 on admission and 3.1±4.0 on discharge, respectively. Three patients did not survive the ischemic event. All of them had a massive ischemic lesion in addition to CC and died 5 days, 11 days, and 16 days after admission, respectively.

The characteristics of the 7 'pure' CC infarction patients are given in Table 2. Patient 1 (Fig. 1A) experienced acute onset of left-side weakness and was noted to have AHS, impaired left limb functions and hemianesthesia. Patient 2 (Fig. 1B) presented with mild ataxia of the left arm as well as slightly reduced strength of lower extremities and reduced tendon reflexes. Babinski's sign (bilaterally) and Chaddock's sign were positive. Patient 3 (Fig. 1C) developed a sudden onset of clumsy speech, blurred vision, unstable walking and headache for 1 day, but no other symptoms. Patient 4 (Fig. 1D) had experienced continuous dizziness for 3 months, which eventually aggravated within 10 days. Neurological dysfunctions comprised mild hemiparesis, hemianesthesia and ataxia. Patient 5 (Fig. 1E) reported to have suffered from vertigo and double vision for 3 days. Neurological examination on admission did not reveal any other pathological signs than diplopia. For patient 6 (Fig. 1F), vertigo and headache for the last 10 days were reported. The patient’s situation worsened rapidly within 12 hours before admission leading to hypoglycemia, right-side weakness and finally coma. Patient 7 (Fig. 1G) showed a very small lesion in the left splenium and presented with mild weakness of lower extremities and slurred speech.

**Table 2:** The characteristics of ‘pure’ and ‘complex’ CC infarctions.

Patient number	1	2	3	4	5	6	7	‘Pure’ CC infarction n = 7	‘Complex’ CC infarction n = 52	*p* value	
Age (years)	58	53	66	74	52	61	78	63.1±10.0	69.1±11.5	0.202	
Sex (M = male, F = Female)	M	F	F	F	F	F	M	2 (28.6)	33 (63.5)	0.109	
Initial NIH Stroke Scale score	1	0	2	2	0	0	1	0.9±0.9	3.3±3.8	**0.027** *	
Discharge NIH Stroke Scale score	0	0	0	2	0	0	0	0.3±0.8	3.5±4.1	**0.002** *	
Hypertension	+	+	+	+	+	+	+	7 (100)	37 (71.2)	0.174	
Atheroma of ICA	+	+	+	+	+	-	+	6 (85.7)	45 (86.5)	1.000	
Dyslipidemia	+	+	+	+	+	-	+	6 (85.7)	26 (50.0)	0.112	
Diabetes mellitus	+	+	+	+	-	+	-	5 (71.4)	24 (46.2)	0.254	
Atheroma of vertebrobasilar artery	+	+	-	+	+	-	-	4 (57.1)	12 (23.1)	0.078	
Heart diseases	+	-	-	-	-	-	-	1 (14.3)	20 (38.5)	0.403	
Heavy drinking	+	-	-	-	-	-	-	1 (14.3)	14 (26.9)	0.666	
Current smoking	+	-	-	-	-	-	-	1 (14.3)	19 (36.5)	0.404	
Prior stroke or TIA	-	-	-	-	-	-	-	0 (0)	14 (26.9)	0.181	
Family history of stroke	-	-	-	-	-	-	-	0 (0)	2 (3.8)	1.000	
Hemiparesis	+	+	-	+	-	-	+	4 (57.1)	39 (75.0)	0.375	
Sensory abnormality	+	-	-	+	-	-	-	2 (28.6)	17 (32.7)	1.000	
Dysarthria	-	-	+	-	-	-	+	2 (28.6)	11 (21.1)	0.643	
Ataxia	-	+	-	+	-	-	-	2 (28.6)	4 (7.7)	0.144	
Alien hand syndrome	+	-	-	-	-	-	-	1 (14.3)	1 (1.9)	0.225	
Disturbance of consciousness	-	-	-	-	-	+	-	1 (14.3)	5 (9.6)	0.548	
Bilateral cerebral hemisphere involvement	-	-	-	-	-	+	-	1 (14.3)	18 (34.6)	0.411	
Aphasia	-	-	-	-	-	-	-	0 (0)	11 (21.1)	0.328	
Etiologic classification, n (%)										
Large-artery atherosclerosis	-	+	-	+	+	-	-	3 (42.9)	31 (59.6)	0.443	
Cardioembolism	-	-	-	-	-	-	-	0 (0)	8 (15.3)	0.578	
Small-vessel occlusion	-	-	-	-	-	+	-	1 (14.3)	4 (7.7)	0.481	
Stroke of other determined etiology	-	-	-	-	-	-	-	0 (0)	1 (1.9)	1.000	
Stroke of undetermined etiology	+	-	+	-	-	-	+	3 (42.9)	8 (15.4)	0.112	
Infarctions of the CC										
Genu	+	+	+	-	-	-	-				
Body	+	+	+	-	+	-	-				
Splenium	+	+	-	+	-	+	+				

TIA = Transient cerebral ischemia. ICA = internal carotid artery. Values are number (%) or mean±standard deviation.

Normally distributed numerical variables: Student’s *t*–test; non-normally distributed numerical variables: non-parametric test (Wilcoxon rank-sum test); categorical variables: chi-squared test.

*p<0.05.

Out of the 7 ‘pure’ CC infarction cases, 1 patient presented infarction of the CC body. Splenium infarctions were found in 3 patients and both genu and body infarctions were diagnosed in 1 patient. In 2 patients, combined genu, body and splenium infarctions were observed, while 1 patient had CC infarction across the midline. The TOAST classification of the 7 ‘pure’ CC infarction cases were: large-artery atherosclerosis (n = 3), small-artery occlusion (n = 1), and undetermined etiology (n = 3).

The most common risk factor was hypertension (n = 7), followed by dyslipidemia (n = 6 each) and diabetes mellitus (n = 5). One patient reported smoking and excessive drinking. One patient was noted to have hypokinetic left ventricular segment. Notably, most of the patients presented with non-specific neurological dysfunctions such as mild hemiparesis (n = 4; muscle strength ranging from 5- to 5 according to the United Kingdom Medical Research Council 6 grades of muscle strength), sensory dysfunction, dysarthria or ataxia (n = 2 each), as well as disturbance of consciousness and AHS (n = 1).

Lesion proportion, neurological dysfunctions and risk factors were compared between the genu and/or body group and splenium group. We observed that there were no differences regarding gender (*p* = 0.778) and age (*p* = 0.398) between these two groups. Comparing the frequency of ICA atheroma in genu and/or body group and vertebrobasilar artery atheroma in splenium group respectively, revealed that genu and/or body infarctions were more frequently diagnosed in patients with atheroma (*p*<0.05). A higher frequency of bilateral cerebral hemisphere involvement was observed in the splenium group (46.2%) as compared to patients with lesions in CC genu and/or body (*p*<0.05).

## Discussion

Epidemiology and pathology of the CC infarction are not well understood so far. Here, we presented a single center, retrospective study of corpus callosal infarction involving 59 patients. Seven patients had pure callosal lesions while the splenium was identified as the most susceptible location for ischemic stimuli insult. In most patients, ischemic lesions were not restricted to the CC and were associated with additional ischemic lesions in the ipsi- and/or contralateral hemisphere. Giroud and Dumas [5] described 282 ischemic stroke patients and concluded that the proportion of CC infarction was 4.6%. Chrysikopoulos and colleagues [10] reported a 7.9% proportion of CC infarction. In our study, only 7 patients (0.43%) presented isolated CC infarctions. The overall low prevalence of CC infarction may be due to the dual CC blood supply from both the anterior and posterior cerebral circulation. The perpendicular orientation of the callosal branches prevents critical embolization of CC-supplying arteries, at least to some extent [10,11]. Another possible reason is that CC is a dense white matter tract which is slightly less sensitive to hypoxia or transient ischemia than gray matter [12]. Hence, transient or chronic hypoxic episodes less often become symptomatic than in comparable grey matter areas.

CC infarctions were previously reported to be most frequently located in the splenium, followed by body and genu [10]. It was assumed that this may result from the greater incidence of infarction in areas with PCA supply. Some authors reported infarctions exclusively in the CC genu and body, but not in the splenium [4]. In contrast, the splenium was the most susceptible location for ischemic stroke in the present study. There was no significant difference in the proportion of ischemic stroke between the genu and/or body and splenium. These controversial data may result from the low incidence of CC infarctions and the lack of large-scale studies. On the other hand, race differences may also play a role. The three studies [4,5,10] we refer to were all conducted in Whites or mixed populations, while this is the first study focusing on CC infarction in an Asian population. It is known that despite a high incidence of stroke in China, whites have more severe (more than 50%) extracranial lesions, while Chinese more frequently suffer from severe intracranial lesions [13]. This may have led to different stroke patterns in our study as compared to Western patient populations. In addition, Chinese have high dietary salt resulting in a high prevalence of hypertension while, at the same time, awareness, treatment and control of hypertension are far from satisfactory in China [14]. This situation is particularly evident in Northeast China which includes the Dalian region [15]. Whether or not this could contribute to the present results remains for further investigation.

Most patients with CC infarctions showed non-specific symptoms. These were restricted vertigo or headache in some cases, which might be a plausible explanation for late hospital admission (up to 10 days after symptom onset) in some cases. In this study, 2 out of 59 patients suffered from AHS (Fig. 1A and H) [16]. Both patients showed complete CC infarction, suggesting that the classical callosal disconnection syndrome occurs predominantly when the CC is severely damaged. On the other hand, AHS may be masked by more severe symptoms, particularly in patients suffering from multiple and/or extensive lesions including those outside the CC. This could also explain why a splenium disconnection syndrome (alexia without agraphia) [17] was not reported in the current study, although we identified 26 splenium infarction patients including 9 with bilateral splenium lesions.

In the observed population, 52 out of 59 patients showed multiple infarctions and 19 presented lesions affecting both hemispheres. Thus, it is plausible that the most likely reason for CC infarction was embolism. However, in cases presenting lesions affecting CC bilaterally (n = 14), anatomical variants in the Circle of Willis or hypoperfusion cannot be excluded as possible mechanisms. Moreover, genu and/or body infarction seemed to be related to large-artery atherosclerosis. This has to be interpreted carefully since carotid artery ultrasound is more sensitive to the plaques in the ICA than in the vertebrobasilar artery and the majority of the patients (51 out of 59) were subjected to carotid artery ultrasound examinations.

Finally, our study comes with a number of limitations, including the retrospective chart review, small sample size, and small number of isolated CC lesions. Most of these are due to the very heterogeneous and often mild neurological dysfunction caused by ischemic CC infarctions, as well as the very low incidence of this rare stroke entity. Accordingly, the exact pathophysiological cascades leading to CC infarction require further detailed investigation which, due to the aforementioned overall low incidence and potential life style or race differences, may call for large, international multi-center approaches.
